# Successful Treatment of Hypothalamic Obesity with Tirzepatide

**DOI:** 10.1016/j.aed.2025.03.004

**Published:** 2025-04-10

**Authors:** Sharmela Brijmohan, Jamie A. Mullally

**Affiliations:** Westchester Medical Center. Endocrinology, Diabetes and Metabolism, School of Medicine, New York Medical College, Valhalla, New York

**Keywords:** hypothalamic, obesity, hypothalamic obesity, tirzepatide, antiobesity medication

## Abstract

**Background/Objective:**

Hypothalamic obesity (HO) is a rare but severe form of obesity characterized by hypothalamic damage resulting in hyperphagia and decreased energy expenditure. Tumors involving the hypothalamus, most commonly craniopharyngiomas, frequently result in HO. Treatment is typically refractory to standard antiobesity treatment modalities. Herein, we describe a young man with HO due to surgical resection of a large craniopharyngioma who was successfully treated with tirzepatide, a novel treatment for obesity and type 2 diabetes.

**Case Report:**

A 21-year-old man presented with headaches for 7 months. His physical examination revealed a body weight of 125 kg (body mass index [BMI], 35 kg/m^2^). Brain magnetic resonance imaging revealed a 5-cm multicystic lobulated sellar mass with suprasellar extension. He underwent transsphenoidal resection, and pathology revealed craniopharyngioma. Postoperatively, he developed hyperphagia and rapid weight gain of 15 kg over 3 months (weight, 140 kg; BMI, 40 kg/m^2^). He was started on tirzepatide, which was gradually up titrated to 10 mg in the first 4 months, during which time he lost 9 kg (weight, 131 kg; BMI, 37 kg/m^2^).

**Discussion:**

The pathophysiology of HO is complex, involving decreased sympathetic activity and energy expenditure, central insulin and leptin resistance, and increased energy storage in adipose tissue. By modulating sympathetic/parasympathetic tone and regulating energy balance, tirzepatide appears to be a promising agent to address the complex pathophysiology of HO.

**Conclusion:**

This case report highlights the novel use of tirzepatide in the treatment of HO. This case informs clinicians of the potential benefits of considering tirzepatide in the management of HO and encourages further exploration of its use in this context.

**Highlights**

• Hypothalamic obesity (HO) is a complex disorder characterized by hyperphagia and excessive weight gain caused by disruptions in the hypothalamic regulation of metabolism and has been historically difficult to treat
• Novel agents such as tirzepatide, a dual glucose-dependent insulinotropic polypeptide/glucagon-like peptide-1 receptor agonist, target multiple weight regulatory pathways disrupted in HO and led to significant weight loss in our patient
• This case highlights the need for further investigation of tirzepatide in managing patients with HO

**Clinical Relevance**

This case report highlights the potential of tirzepatide in treating hypothalamic obesity (HO), a rare and challenging condition. Although advances in modern treatments have greatly improved survival rates for patients with craniopharyngiomas, their health is often significantly impaired by severe obesity. The potential for significant weight loss with tirzepatide offers hope for a new therapeutic approach for individuals affected by HO.

## Introduction

The hypothalamus plays a crucial role in regulating energy homeostasis and appetite. Hypothalamic obesity (HO) is a rare but severe form of obesity that arises from damage to the hypothalamus, leading to hyperphagia and decreased energy expenditure.^1^ This condition has been linked to tumors of the central nervous system, particularly craniopharyngioma, and can result from direct tumor effects or surgical and radiotherapeutic interventions. Notably, approximately 50% to 75% of patients treated for craniopharyngioma will develop HO.^2^^,^^3^

Previous studies have demonstrated the difficulty in treating HO, with limited success using dietary, physical activity, and behavioral interventions. Pharmacotherapeutic and bariatric surgical approaches in the past have also shown limited effectiveness.

In this case report, we describe a young man with HO following the surgical resection of a large craniopharyngioma, who was successfully treated with tirzepatide—a novel treatment for obesity and type 2 diabetes. This case highlights the potential of tirzepatide as a promising therapeutic approach for managing this complex condition.

## Case Report

**Fig. 1:**
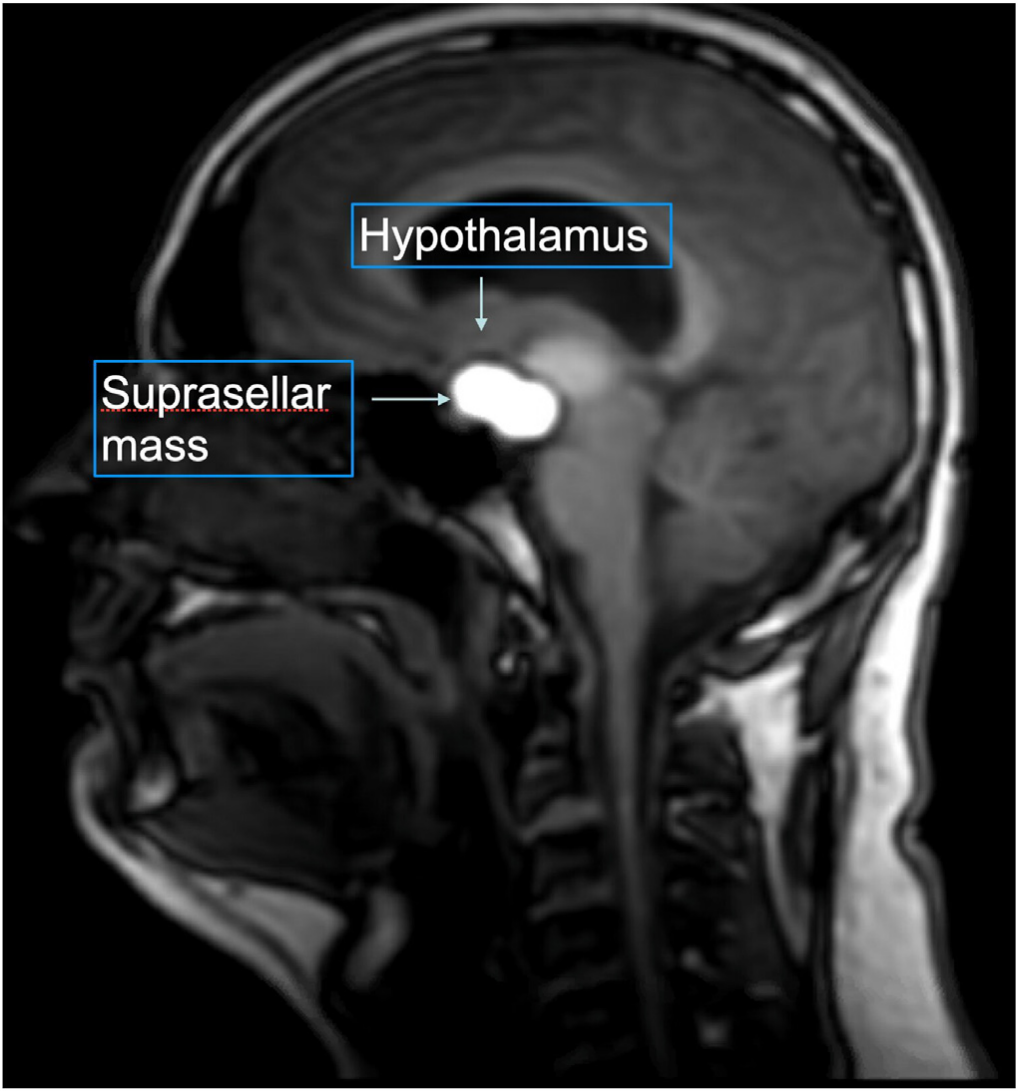
Magnetic resonance Images of the brain without and with intravenous gadolinium using multiplanar multisequence imaging. Large, predominantly cystic sellar and suprasellar mass measuring approximately 4.7 × 2.7 × 3.6 cm (anteroposterior × transverse × craniocaudal), with enhancing solid components and multiple fluid-filled levels, including layering hemorrhage. Mass effect was exerted upon surrounding structures, including the optic chiasm, third ventricle, bilateral posterior and anterior cerebral arteries, as well as the right cavernous and supraclinoid internal carotid artery. Hydrocephalus with mild periventricular T2/fluid-attenuated inversion recovery hyperintensity.

A 21-year-old previously healthy man with obesity presented with a 7-month history of headaches. On physical examination, he was found to have generalized obesity with a body weight of 125 kg (body mass index [BMI], 35 kg/m^2^). He did not appear cushingoid or acromegalic. Table 1 shows the laboratory results. Brain magnetic resonance imaging with and without contrast revealed a large, multicystic, lobulated sellar mass measuring approximately 4.7 × 2.7 × 3.6 cm (anteroposterior × transverse × craniocaudal), with extensive suprasellar extension (Fig. 1).

**Table 1:** Laboratory Results on Admission Prior to Surgery

Test	Result	Reference range
Prolactin	23 ng/mL	3-18 ng/mL
TSH	3.7 mIU/L	0.3-4.7 mIU/L
FT4	1.3 ng/dL	0.8-1.8 ng/dL
Morning cortisol	21 mcg/dL	3.7-19 mcg/dL
LH	2.6 mIU/mL	0.6-12 mIU/mL
FSH	2.8 mIU/mL	1.4-13 mIU/mL
Total testosterone	320 ng/dL	240-950 ng/dL
IGF-1	166 ng/mL	91-442 ng/mL
Hemoglobin A1c	5%	<5.7%

Abbreviations: FSH = follicle-stimulating hormone; FT4 = free thyroxine; IGF-1 = insulin-like growth factor 1; LH = luteinizing hormone; TSH = thyroid-stimulating hormone.

The patient underwent transsphenoidal resection, and pathology revealed an adamantinomatous craniopharyngioma, central nervous system World Health Organization grade 1. Following surgery, the patient developed panhypopituitarism and was initiated on hormonal replacement therapy with desmopressin, levothyroxine, hydrocortisone, and testosterone. However, he soon developed hyperphagia and rapid weight gain of 15 kg in 3 months, with an increase in weight from 125 kg (BMI, 35 kg/m^2^) to a weight of 140 kg (BMI, 40 kg/m^2^). Diet and physical activity were recommended during this period; however, he continued to gain weight. He was then started on tirzepatide at a dose of 2.5 mg weekly, which was gradually uptitrated to 10 mg weekly over 4 months.

**Fig. 2:**
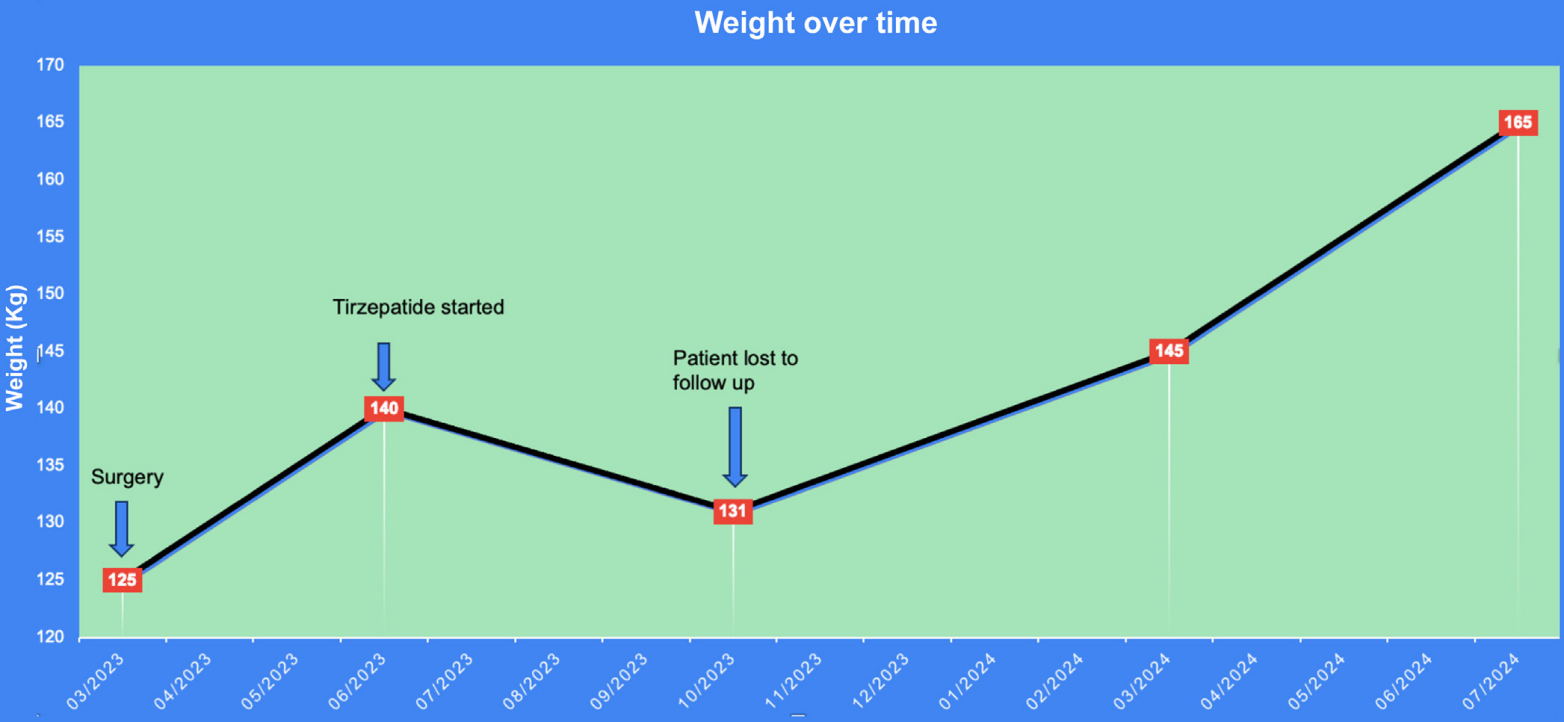
The patient underwent surgery on March 2023 with a baseline weight of 125 kg (body mass index [BMI], 35 kg/m^2^). Postoperatively, his weight increased to 140 kg over 3 months (BMI, 40 kg/m^2^) despite diet and exercise. He was started on tirzepatide 2.5 mg weekly on June 2023, which was uptitrated to 10 mg over 4 months. On treatment, his weight was reduced to 131 kg (BMI, 37 kg/m^2^). He was then lost to follow-up and stopped tirzepatide, and he gained to 165 kg over 9 months (BMI, 47 kg/m^2^).

After starting tirzepatide, he reported a lower appetite and earlier satiety. Over 4 months, the patient gradually lost 9 kg, reducing his weight to 131 kg (BMI, 37 kg/m^2^). The patient did not report any side effects from tirzepatide and tolerated the dose escalation well. However, he was lost to follow-up and has stopped the medication. Despite his loss to follow-up, we were able to obtain data on his weight, as he continues to see other providers within our electronic medical record system. The significant distance from his residence to our endocrine practice, along with a recent worsening of his depression, may have affected his ability to continue care with us. Since discontinuing tirzepatide, a rapid weight gain of 34 kg, to now 165 kg (BMI, 47 kg/m^2^) over 9 months, with a continued upward trajectory has been observed (Fig. 2).

## Discussion

This case report describes a patient with acquired HO resulting from surgical treatment of a large craniopharyngioma. HO is a condition characterized by rapid and uncontrollable weight gain, unresponsive to diet and exercise, and often accompanied by hyperphagia and abnormal food-seeking behaviors.^4^

Craniopharyngiomas are benign yet locally aggressive tumors that are frequently implicated in the etiology of HO. These neoplasms may grow slowly in the suprasellar region and can invade surrounding tissue including the hypothalamus.^5^

Notably, the patient described exhibited significant obesity before surgical intervention, suggesting some degree of preexisting hypothalamic dysfunction due to the tumor’s suprasellar extension. In HO, the location of the primary tumor is one of the principal factors affecting body weight and composition.^4^ In a large study of 63 children, 17% had a history of significant weight gain in the 3.5 years before the diagnosis of craniopharyngioma.^6^

Postoperatively, the patient experienced hyperphagia and rapid weight gain, which is likely attributable to additional damage to the hypothalamic-pituitary axis incurred during surgical resection of the craniopharyngioma. After tumor resection, survivors of childhood craniopharyngioma are known to experience an increased rate of approximately 50% to 75% of obesity.^2^^,^^3^

The most substantial weight gain in HO typically occurs during the first 12 months after surgery. This rapid postoperative weight gain is also a predictor for severe long-term obesity. The pathophysiology of HO is characterized by impairment in the key brain pathways that regulate energy intake and expenditure, autonomic nervous system function, and peripheral hormonal signaling.^3^

Damage to the medial hypothalamic nuclei caused by a tumor, surgery, or radiation disrupts feeding circuits. This causes the appetite-stimulating signals in the lateral hypothalamus to become overactive, whereas the appetite-suppressing pathways in the medial hypothalamus are weakened, leading to excessive food intake.^2^^,^^4^

Damage to the medial hypothalamus can also lead to disruption of vagal tone, resulting in excess stimulation of pancreatic β-cells and hyperinsulinemia. Sympathetic nervous output is reduced, leading to decreased resting energy expenditure and less voluntary physical activity. Additionally, lower energy expenditure via reduced fatty acid oxidation in the peripheral tissues may be due to reduced serum α-melanocyte-stimulating hormone, a hypothalamic hormone from the melanocortin pathway.^4^

Hypothalamic damage also affects the arcuate nucleus, the main binding site for peripheral hormones. This prevents the hypothalamus from properly responding to adiposity and satiety signals such as insulin, leptin, and gut hormones, including glucagon-like peptide-1 (GLP-1).^1^^,^^2^

HO has been particularly resistant to treatment, with most modalities failing to achieve significant weight loss and showing varied responses among participants. Additionally, given the rarity of the condition, most trials have been too small to determine effectiveness. Table 2^7^, ^8^, ^9^, ^10^, ^11^ provides an overview of different drug treatments for HO.

**Table 2:** Prior Treatments for Hypothalamic Obesity and Outcomes

Prior treatments for hypothalamic obesity	Outcomes
Lifestyle interventions	Lifestyle interventions only resulted in BMI decrease in the short term.^7^ It is a therapeutic cornerstone but often fails to result in meaningful and sustained reduction in BMI.^8^
Orlistat, a pancreatic lipase inhibitor	Rarely used due to low efficacy and GI side effects.^7^
Diazoxide, an inhibitor of glucose-stimulated insulin release	No difference in weight when compared with placebo.^9^
Metformin	Combined with diazoxide has shown slightly more promising results in slowing weight gain albeit not leading to weight loss.^9^
Central stimulants (methylphenidate, phentermine, dextroamphetamine, mazindol, caffeine, and ephedrine)	Data on these drugs came from small studies with mixed results.^7^
Sibutramine, a norepinephrine and serotonin inhibitor	Shown to promote weight loss but discontinued due to safety concerns.^7^
Octreotide, a somatostatin analog	A double-blind, placebo-controlled study on octreotide therapy for pediatric hypothalamic obesity demonstrated significantly reduced weight gain compared with placebo. However, use is limited due to high cost and the limited availability of long-term data.^10^
Bariatric surgery including Roux-en-Y gastric bypass, gastric banding, and sleeve gastrectomy	Meta-analysis results indicate that bariatric surgery significantly reduces weight in patients with hypothalamic obesity but its effectiveness is notably lower than in those with common obesity. This is likely because hypothalamus feedback integrity is necessary for effective bariatric surgery.^11^

Abbreviations: BMI = body mass index; GI = gastrointestinal.

Tirzepatide, a novel agent approved by the Food and Drug Administration for the treatment of obesity and type 2 diabetes, is a dual glucose-dependent insulinotropic polypeptide and GLP-1 receptor agonist. It binds to GLP-1 receptors in the hindbrain, activating satiety signaling pathways in a region that remains functional even in individuals with severe hypothalamic damage. Additionally, its action on glucose-dependent insulinotropic polypeptide receptors enhances its antiobesity effects by regulating energy balance through cell surface receptor signaling in adipose tissue and the central nervous system, potentially counteracting the deficient melanocortin pathway in the hypothalamus, which plays a central role in the regulation of energy homeostasis.^2^^,^^12^

Induction of weight loss is believed to be related to targeting multiple redundant weight regulatory pathways involving the gastrointestinal tract, autonomic nervous system, and hypothalamus.^12^

Given the rapid weight gain despite efforts with dietary modification and increasing physical activity, our patient was started on tirzepatide. A 72-week, double-blind, randomized, controlled trial of 2539 adults, with a BMI of at least 27 kg/m^2^, demonstrated substantial and sustained weight reductions in participants with obesity receiving tirzepatide (5 mg, 10 mg, or 15 mg once weekly).^13^ At baseline, the mean body weight was 104.8 kg. The mean percentage changes in weight at week 72 were −15.0% with 5-mg weekly doses of tirzepatide, −19.5% with 10-mg doses, and −20.9% with 15-mg doses compared with −3.1% with placebo (*P* < .001). However, patients with HO were excluded from this and other landmark trials of tirzpeatide.^13^ Remarkably, our patient with HO achieved a significant weight loss of 9 kg (−6.4%) over 4 months at a fairly low dose of tirzepatide (2.5-10 mg).

Several studies have evaluated the use of semaglutide for treating HO. A case report by Sciacovelli et al^14^ described a patient who lost over 30 kg after 6 months of semaglutide therapy, uptitrated to 2 mg per week. Similarly, a case series by Gjersdal et al^15^ found a mean weight loss of 20 kg over 6 months in patients with craniopharyngioma, with doses increased to their maximally tolerated levels (1.7-2.4 mg per week). Additionally, Svendstrup et al^16^ studied semaglutide treatment in 26 patients with HO, reporting a mean weight loss of 13.4 kg after 12 months, with a median dose of 1.6 mg (range, 0.5–2.5 mg).

In our case report, the patient gradually lost 9 kg on tirzepatide, titrated from 2.5 mg to 10 mg over 4 months. Similar to studies with semaglutide, our findings suggest that tirzepatide offers promising results for treating HO. However, it is important to note that our findings were limited to treatment with tirzepatide for only 4 months at a less than maximal dose so we would expect greater weight loss with treatment for a longer duration and with a higher dose of tirzepatide.

A cohort study by Rodriguez et al^17^ compared weight loss outcomes between patients receiving tirzepatide and semaglutide for overweight or obesity. Their findings indicate that individuals treated with tirzepatide were significantly more likely to achieve greater reductions in body weight than those receiving semaglutide.^17^

Postoperatively, our patient reported poor sleep, extreme fatigue, and depressed mood. A study by Sterkenburg et al^18^ reported that the quality of life in craniopharyngioma patients with hypothalamic involvement is impaired by obesity, physical fatigue, reduced motivation, dyspnea, diarrhea, and suboptimal psychosocial development. This highlights the significant burden of hypothalamic dysfunction on overall well-being.

## Conclusion

In summary, tirzepatide appears to be a promising treatment for the often refractory weight gain seen in patients with HO. Further study is needed to expand upon and confirm our results in other patients. This case underscores the importance of continued investigation into the pathophysiology of HO and the need for effective treatments for this debilitating condition.

## Disclosure

The authors have no conflicts of interest to disclose.
